# Key factors selection on adolescents with non-suicidal self-injury: A support vector machine based approach

**DOI:** 10.3389/fpubh.2022.1049069

**Published:** 2022-11-10

**Authors:** Jiaxin Yang, Yinghao Chen, Gongyu Yao, Zheng Wang, Xi Fu, Yusheng Tian, Yamin Li

**Affiliations:** ^1^Clinical Nursing Teaching and Research Section, The Second Xiangya Hospital, Central South University, Changsha, China; ^2^Department of Emergency Medicine, The Second Xiangya Hospital, Central South University, Changsha, China; ^3^Department of Cardiovascular Surgery, The Second Xiangya Hospital, Central South University, Changsha, China; ^4^Eastern Institute for Advanced Study, Yongriver Institute of Technology, Ningbo, China; ^5^School of Mathematics and Statistics, Central South University, Changsha, China; ^6^National Clinical Research Center for Mental Disorders, Department of Psychiatry and Hunan Medical Center for Mental Health, The Second Xiangya Hospital of Central South University, Changsha, China

**Keywords:** non-suicidal self-injury, mood disorders, artificial intelligence, support vector machine, adolescents

## Abstract

Comparing a family structure to a company, one can often think of parents as leaders and adolescents as employees. Stressful family environments and anxiety levels, depression levels, personality disorders, emotional regulation difficulties, and childhood trauma may all contribute to non-suicidal self-injury (NSSI) behaviors. We presented a support vector machine (SVM) based method for discovering the key factors among mazy candidates that affected NSSI in adolescents. Using SVM as the base learner, and the binary dragonfly algorithm was used to find the feature combination that minimized the objective function, which took into account both the prediction error and the number of selected variables. Unlike univariate model analysis, we used a multivariate model to explore the risk factors, which better revealed the interactions between factors. Our research showed that adolescent education level, anxiety and depression level, borderline and avoidant personality traits, as well as emotional abuse and physical neglect in childhood, were associated with mood disorders in adolescents. Furthermore, gender, adolescent education level, physical abuse in childhood, non-acceptance of emotional responses, as well as paranoid, borderline, and histrionic personality traits, were associated with an increased risk of NSSI. These findings can help us make better use of artificial intelligence technology to extract potential factors leading to NSSI in adolescents from massive data, and provide theoretical support for the prevention and intervention of NSSI in adolescents.

## Introduction

Non-suicidal self-injury (NSSI) is a common psychiatric behavioral problem that threatens adolescents' health. NSSI refers to behaviors that do not aim at suicide, intentionally and directly harm their body tissues and are not recognized by society and culture (1). Common types of NSSI include cutting, scratching, hitting, knocking, and burning themselves (1). In 2013, the Diagnostic and Statistical Manual of Mental Disorders (5th edition) (DSM-5) classified NSSI as a separate disorder and established strict diagnostic criteria. Currently, suicide has become the third leading cause of death among adolescents (10–19 years old) worldwide, and NSSI is an important prerequisite for adolescent suicide and one of the strongest predictors of future suicide (2), adding a huge medical and economic burden to society. Furthermore, NSSI is associated with other adverse outcomes, such as cognitive impairment, poor interpersonal relationships, and violent crimes (3). NSSI has become an important global adolescent mental health problem. Currently, there are still many clinical challenges in the identification and intervention of NSSI (4). However, understanding the factors associated with NSSI is essential for conducting clinical risk assessment and therapeutic interventions.

The incidence of NSSI is higher in adolescents than in any other age group (5). Lim et al. (6) conducted a systematic review of 66 studies and showed that the lifetime and 12-month incidence of NSSI were 22.1 and 19.5%, respectively. In addition, there were differences in the incidence of NSSI among different regions and ethnic groups. Studies have shown that Caucasians have the highest incidence of NSSI (7). Zubrick et al. (8) surveyed 2,967 adolescents in Australia in 2013–2014 and found that the 12-month incidence of NSSI in adolescents was about 8%. Brunner et al. (9) investigated 12,068 adolescents from 11 countries in Europe in 2014 and found that the lifetime incidence of self-injury was 27.6%, ranging from 17.1 to 38.6% in each country. A recent meta-analysis study showed that the 12-month incidence of NSSI among adolescents was higher in low- and middle-income countries than in high-income countries (6). In recent years, the incidence of NSSI among adolescents in China has been on the rise, and surveys in different cities and regions have shown that the prevalence of NSSI among adolescent ranges from 5.4 to 33.8% (10). Moreover, the onset age of adolescent NSSI behavior is mainly concentrated in early adolescence (12–14 years old). The incidence of NSSI peaks in mid-adolescence (15–16 years old), and then decreases in late adolescence (18 years old) (11). Notably, the early onset of NSSI creates greater disease vulnerability. Studies have shown that individuals with NSSI onset younger than 12 years old tend to develop severe NSSI behaviors (12). Moreover, NSSI in early adolescence may predict the onset of mental disorders in late adolescence. Therefore, identifying risk factors for NSSI in adolescents is important for the early identification and prevention of NSSI behaviors.

Earlier studies have shown that NSSI behavior is the end product of interactions between genetic, biological, psychiatric, psychological, social and cultural factors (13). The potential causes of NSSI include individual and environmental factors (14). Individual factors include mental illness, personality traits, and emotional regulation abilities. Depressive disorder and borderline personality disorder are the most common co-morbidities in the adolescent with NSSI. Depressive symptoms are important predictors of NSSI behavior in adolescents (15). In addition, NSSI is considered to be a precursor of borderline personality disorder in a socio-biological developmental model (16). NSSI behaviors are associated with adolescents' personality traits. Studies have shown that adolescents with NSSI show higher levels of impulsivity than those without NSSI (17) and have higher scores on all subscales of the Barratt Impulsivity Scale (18). Furthermore, emotional regulation plays an important role in adolescents' NSSI behavior (19). On the other hand, the family environment is the most important growth environment for adolescents and plays an important role in the development of adolescents' characters and behaviors. Family systems theory pointed out that family functions affected the physical and mental health of family members (20), and had an important impact on individual emotional and behavioral problems (21). Research indicated that poor family function mediated the link between childhood adversity and NSSI in adolescents (22). In addition, adolescents with pathological family relationships and disharmonious relationships with their parents have a higher risk of self-harm (23). Furthermore, experiencing adverse life events (parental abuse or neglect) in childhood (24), parental divorce (25), family death (26), and witnessing parental violence (27) have all been associated with NSSI behaviors in adolescents. Notably, improving family relationships may reduce NSSI in later childhood who experience adverse life events in childhood. Therefore, understanding the family environment of Chinese adolescents with NSSI is beneficial to explore the influence of family factors on adolescents with NSSI, thereby improving family relationships and functions.

Feature selection is a key preprocessing mechanism in data mining tasks, which avoids the interference of irrelevant variables by finding the optimal feature subset from the given dataset (28). With the development and enrichment of artificial intelligence technology, researchers have developed a large number of feature selection methods (29), including three categories of filter (30), wrapper (31), and embedding (32). The ensemble algorithm based on the decision tree model, such as random forest (33), evaluates the importance of features according to the improvement of purity. Principal component analysis (34, 35) and its improvements (36) are also commonly used methods of dimensionality reduction. The feature is transformed into a set of line-independent variables through orthogonal transformation, so that a large number of variables can be represented by a few principal components. The least absolute shrinkage and selection operator (LASSO) (37, 38) and ridge regression methods (39) take the features' weight as a regularization term while considering the accuracy of multiple linear regression to reduce the number of selected features. In this paper, inspired by the LASSO method, we proposed a support vector machine (SVM) based feature selection method to find the key indicators of NSSI. We used a binary state vector to represent the selected indicator, took the average error rate of the SVM classifier 10-fold cross-validation as the objective function, and the other part of the objective function was the number of selected indicators. The binary dragonfly algorithm was used to optimize the state vector to minimize the objective function.

Currently, most studies on adolescent NSSI in China have explored the effect of a single factor on NSSI behavior or explored the correlation between two factors, and no studies have yet analyzed the effect of individual and family factors on adolescent NSSI behavior. Thus, we present a support vector machine (SVM) based method to discover the key factors among individual and family factors influencing adolescent NSSI, providing a theoretical basis for the prevention and intervention of adolescent NSSI.

## Methods

### Participants

We conducted this cross-sectional research from June 2020 to April 2021 in China. Adolescents aged 10–24 years were recruited. Rather than age 10–19 years, the definition of 10–24 years corresponds more closely to adolescent growth and the general understanding of this life stage, facilitating expanding research (40). What's more, an expanded and broader definition of the adolescent is critical to the development of social policies and service systems (40). Adolescents diagnosed with mood disorders (i.e., depressive disorder, depressive episode of bipolar disorder, and unspecified behavioral and mood disorders originating in childhood and adolescence) according to the International Classification of Diseases-10 (ICD-10) were recruited from the psychological ward and outpatient clinic of a tertiary hospital in Changsha, China. After eliminating participants with suicidal ideation and attempts within the past 12 months, participants with mood disorders were divided into two groups based on the DSM-5 criteria of NSSI: with and without NSSI groups. Typical developmental (TD) adolescents studying in primary school, middle school, or university were recruited. All participants or parents signed an informed consent form. Adolescents were excluded if they: (1) suffered from severe somatic disorders; (2) had cognitive impairment, audiovisual impairment, etc.; (3) suffered from other severe mental illnesses. The research was approved by the medical ethics committee of the Second Xiangya Hospital of Central South University (MD20200309).

### Measures

#### Sociodemographic information

The surveys included self-reported information on sociodemographic characteristics including adolescents' characteristics (i.e., gender, age, ethnicity, education level, grade) and parental characteristics (i.e., education level, age).

#### Generalized anxiety disorder-7

The generalized anxiety disorder-7 (GAD-7), developed by Spitzer et al. (41), is used to assess subjects' anxiety symptoms and severity during the last 2 weeks. The scale contains seven items and uses a four-point Likert scale scored from 0 (not at all) to 3 (almost every day). The higher the score, the more severe the anxiety symptoms. A total score of 0–4 indicates no anxiety; 5–9 indicates mild anxiety; 10–13 indicates moderate anxiety; 14–18 indicates moderate to severe anxiety; and 19–21 indicates severe anxiety. The Cronbach's alpha co-efficient for this scale in this study was 0.924.

#### Patient health questionnaire-9

The patient health questionnaire-9 (PHQ-9), developed by Kroenke et al. (42), is used to assess subjects' depressive symptoms during the last 2 weeks. The scale contains 9 items, each of which consists of four options and is scored on a four-point scale (0 = not at all, 3 = almost every day). The higher the score, the more severe the depression. A total score of <5 means no depression, 5–9 mild depression, 10–14 moderate depression, 15–19 moderate to severe depression, and ≥20 severe depression. The Cronbach's alpha co-efficient of internal consistency was 0.927.

#### Personality diagnostic questionnaire-4+

The personality diagnostic questionnaire-4+ (PDQ-4+), developed by Hyler et al. (43), is a self-administered questionnaire for screening personality disorders, which was consistent with the DSM-IV criteria for the 10 officially recognized and two proposed Axis II personality disorders. Yang, a Chinese scholar (44), translated the PDQ-4 into Chinese. Moreover, another Chinese scholar translated the PDQ-4+ into Chinese and revised it for the Chinese cultural context, resulting in a Chinese version of the PDQ-4+ with good reliability and validity (45). In this study, we used the Chinese version of the PDQ-4+ (45). The higher the score, the more consistent the personal characteristic description. The Cronbach's alpha co-efficient of this scale in the present study was 0.921.

#### Difficulties in emotion regulation scale

The difficulties in emotion regulation scale (DERS), developed by Gratz and Roemer in 2004 (46), is a self-report questionnaire used to assess subjects' difficulties in emotion regulation. The scale contains 36 items and six subscales (non-acceptance of emotional responses, difficulties in engaging in goal-oriented behaviors, difficulties in controlling impulses, lack of emotional awareness, restricted access to emotional regulation strategies, and lack of emotional clarity). The scale uses a five-point Likert scale scored from 1 (almost never) to 5 (almost always). The higher the score, the more difficult to regulate emotion. The Cronbach's alpha co-efficient for this scale in this study was 0.941.

#### Childhood trauma questionnaire-short form (CTQ-SF)

Subjects' trauma experience in childhood was assessed by childhood trauma questionnaire-short form (CTQ-SF), which was developed by Bernstein et al. (47) and translated into Chinese by Fu et al. (48). The scale represents a 28-item retrospective self-report questionnaire which contains five subscales: emotional (EN) and physical neglect (PN), emotional (EA), sexual (SA) and physical abuse (PA) (49). Each item is scored on a five-point Likert scale from 1 (never true) to 5 (very often true). The Cronbach's alpha co-efficient of this scale in the present study was 0.793.

### Statistical analysis

#### Univariate analyzes

Multiple groups were compared using Chi-square tests. Meanwhile, non-parametric Kruskal-Wallis tests and pairwise comparisons were used. α < 0.05.

#### Multivariate analysis

##### Related works

Data mining and data analysis through information technology to obtain potential value can better guide people's production and life. In order to reduce the noise contained in the data and improve the reliability of data mining, lots of feature selection algorithms have been proposed and adopted for diagnosis, classification, and categorization in recent years. The tree model computes variable importance by finding the optimal partitioning features that vary purity, information gain, or gain rate, and leads to different decision tree generation schemes, such as iterative dichotomy 3 (ID3) (50), C4.5 (51, 52) and Classification and Regression Trees (CART) (53). Our previous work proposed the mixed correlation co-efficient to measure the linear or non-linear correlation between two variables and used it to study the user participation mechanism in virtual tourism communities (54). As a common tool for big data mining, machine learning algorithms have been more and more widely used in the clinical and medical fields, such as COVID-19 (55–57), Crohn's disease (58), and schizophrenia diagnosis (59–61).

##### Support vector machine

**Figure 1:**
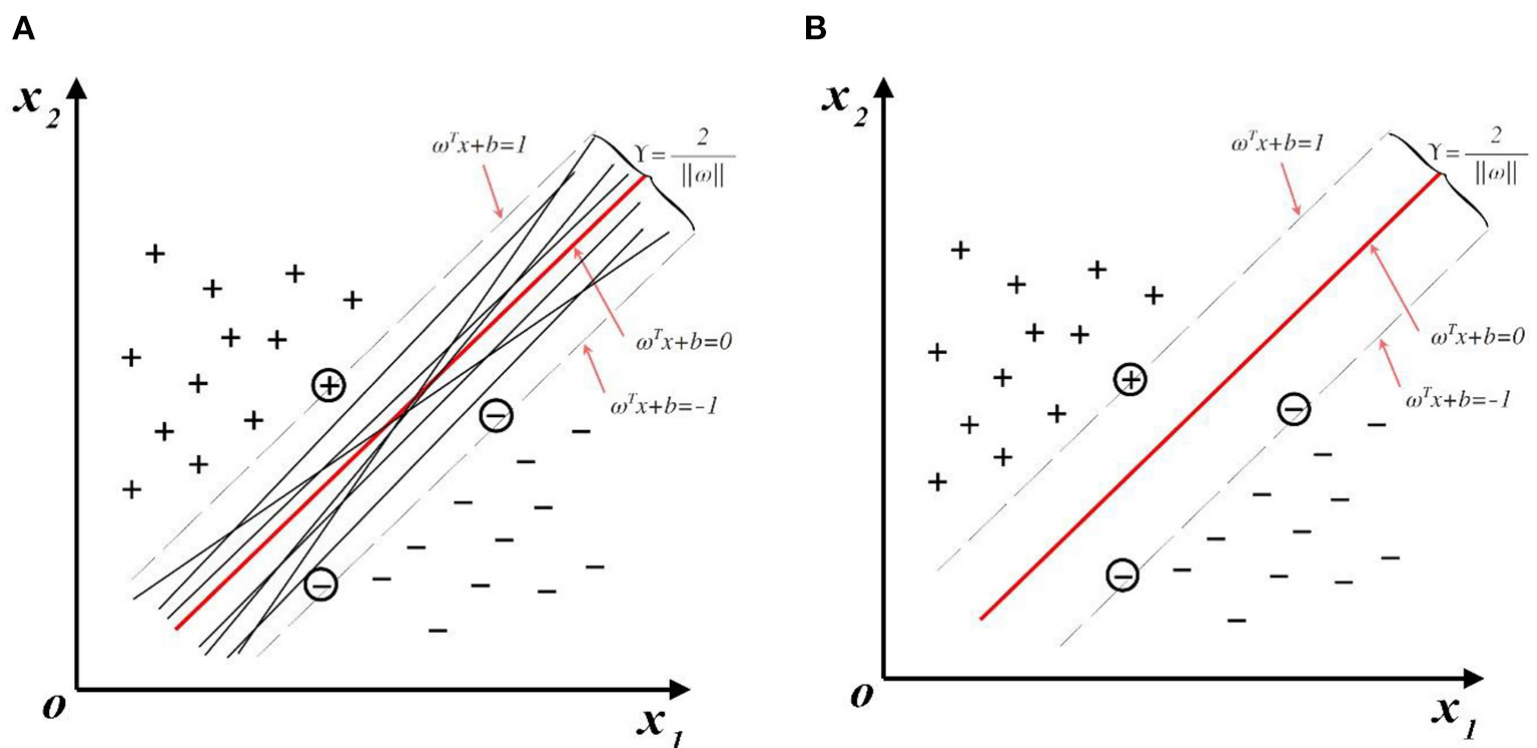
Hyperplanes in sample space: **(A)** There are multiple hyperplanes in the sample space that can accurately classify samples; **(B)** The optimal division hyperplanes.

Support vector machine (SVM) was proposed by Cortex and Vapnik (62) in the 1990s, and quickly became the mainstream technology in machine learning methods due to its excellent performance in text classification. SVM can avoid the curse of dimensionality and overfitting in solving pattern recognition tasks with a small number of samples, non-linearity, and high dimensionality. The principle of support vector classification (SVC) is to find an optimal classification hyperplane, that is, it can tolerate the local disturbance of training samples while distinguishing samples of different categories. As shown in Figure 1A, although multiple hyperplanes can distinguish samples, only the red hyperplane located in the middle of the two classes has the best robustness and is considered the optimal division hyperplane. For the training set {*x*_*i*_, *y*_*i*_} record the positive sample as *y*_*i*_ = 1 and the negative sample as *y*_*i*_ = −1, then the hyperplanes (ω, *b*) is required to hold the following equations:


(1) $$\left\{ \begin{array}{c} \omega^{T}x_{i}+b\geq +1,y_{i}=+1\\ \omega^{T}x_{i}+b\leq -1,y_{i}=-1 \end{array},\right.$$

and the optimal division hyperplanes in Figure 1B can be written as $\omega^{T}x_{i}+b=0$. The optimization goal is to maximize the distance $\gamma =\frac{2}{∥\omega ∥}$ between the support samples and the hyperplane larger, that is:


(2) $$\begin{array}{l} \underset{\omega ,b}{\max}\;\gamma \;,\;such\;that\;y_{i}\left(\omega^{T}x_{i}+b\right)\geq 1,i=1,2,\ldots ,m. \end{array}$$

**Figure 2:**
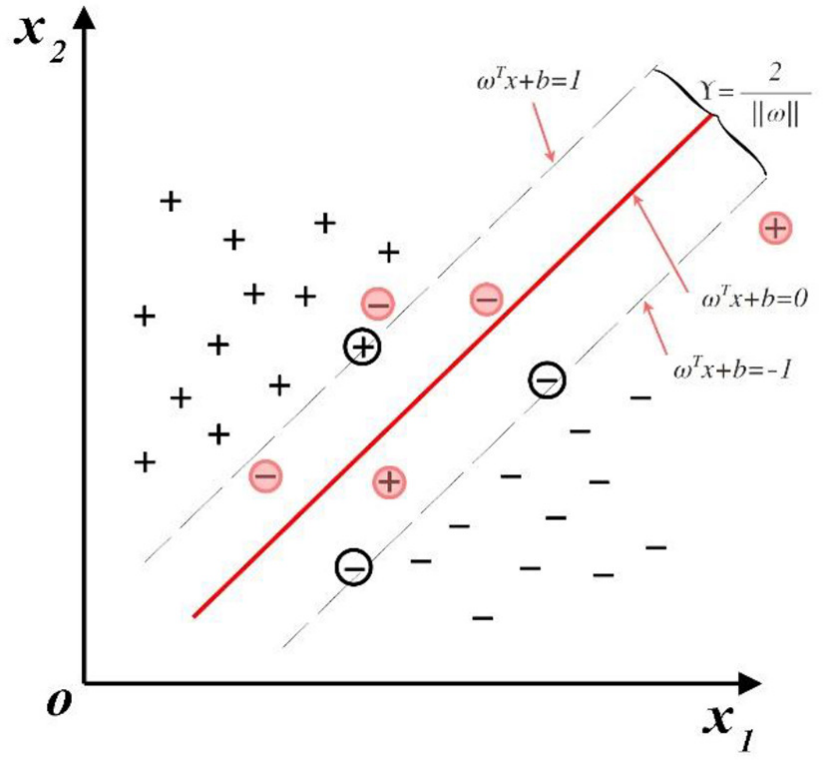
Support vector and optimal hyperplane with slack variable.

In fact, it is difficult to find a hyperplane that can accurately divide the two classes into practical problems. In Figure 2, a slack variable can be introduced to allow SVC to make misclassification of a few samples, and the corresponding optimization function is given by:


(3) $$\begin{array}{l} \underset{\omega ,b}{\min}\frac{∥\omega {∥}^{2}}{2}+C\sum_{i=1}^{m}\max(0,1-y_{i}(\omega^{T}x_{i}+b)), \end{array}$$

Where $||\omega ||=\sqrt{\sum \omega_{i}^{2}}$ is the *l*^2^ norm, and *C* is the penalty co-efficient, The larger *C* is, the fewer training samples do not satisfy the constraints. Equation (3) is equivalent to Equation (2) when *C* → ∞.

##### Binary dragonfly algorithm

The Dragonfly Algorithm (DA) (63) is an intelligent optimization algorithm that finds the optimal solution of the objective function by imitating the group behavior of dragonflies to find food and avoid natural enemies. The binary Dragonfly Algorithm (BDA) is the discrete form of DA that represents the position of the dragonfly as a vector with elements 0 or 1 (64). The behavior of a dragonfly colony includes five main behaviors: namely separation, alignment, cohesion, attraction, and distraction, which are used to update the position of each dragonfly. The mathematical model of these behaviors can be described as follows:

***Separation***. The purpose of separation is to prevent adjacent dragonflies from colliding, that is, two dragonflies are not allowed to exist in the same position at the same time. Thus, we have:


(4) $$\begin{array}{l} S_{i}=-\overset{M}{\underset{j=1}{\sum }}\left(X-X_{j}\right), \end{array}$$

where *X* is the position of a dragonfly in feature space, and there are *M* dragonflies nearby, and their coordinates are marked as *X*_*j*_.

***Alignment***. Alignment requires that the velocity of each dragonfly match its swarm or sub-swarm, preventing falling behind or leaving the swarm. That is, velocity *V* should equal the average speed of the nearby dragonflies:


(5) $$\begin{array}{l} V_{i}=\frac{1}{M}\overset{M}{\underset{j=1}{\sum }}V_{j}, \end{array}$$

***Cohesion***. Cohesion prevents the group from being dissolved, it allows dragonflies to move toward the center of the group, that is:


(6) $$\begin{array}{l} C_{i}=\frac{1}{M}\sum_{j=1}^{M}\left(X-X_{j}\right), \end{array}$$

***Attraction***. Dragonflies need to eat in order to survive, so they are attracted to food. At the same time, the dragonfly in the swarm flew toward the leading dragonfly *X*_*pb*_ closest to the food *X*_*f*_. This process can be defined as:


(7) $$\begin{array}{l} F_{i}=\frac{1}{2}\left[\left(X_{pb}-X_{i}\right)+\left(X_{f}-X_{i}\right)\right], \end{array}$$

***Distraction***. In order to avoid predation, dragonflies need a distraction to stay away from natural enemies. Similar to the attraction process, *X*_*pw*_ means the position of the dragonfly closest to the enemy *X*_*e*_ is also considered. Distraction can be expressed mathematically as:


(8) $$\begin{array}{l} E_{i}=\frac{1}{2}\left[\left(X_{pw}+X_{i}\right)+\left(X_{f}-X_{e}\right)\right], \end{array}$$

We use the iterative algorithm defined in Equation 9 to update the coordinate vector of the dragonfly to guide the dragonfly to find the food, and Δ*X*_*i*_ represents the stepping direction.


(9) $$\begin{array}{l} X_{i}^{d}\left(t+1\right)=\{\begin{array}{c} 1-X_{i}^{d}\left(t\right),\;rand<TF\left(X_{i}^{d}\left(t+1\right)\right)\\ X_{i}^{d}\left(t\right)\;,\;rand\geq TF\left(X_{i}^{d}\left(t+1\right)\right) \end{array}\\ X_{i}^{d}\left(t+1\right)=\alpha_{1}S_{i}+\alpha_{2}V_{i}+\alpha_{3}C_{i}+\alpha_{4}F_{i}+\alpha_{5}E_{i},\\ \;TF\left(\text{Δ}X\right)=\frac{\left|\text{Δ}X\right|}{\sqrt{\text{Δ}X^{2}+1}}, \end{array}$$

where $X_{i}^{d}$ represents the *d*-th position of the *i*-th dragonfly, *rand* is a random number uniformly distributed in the interval [0, 1]. α_*i*_, *i* = 1, 2, 3, 4, 5 are the weights of five behaviors, which are set as 0.2 as default.

##### SVM-based feature selection method

**Figure 3:**
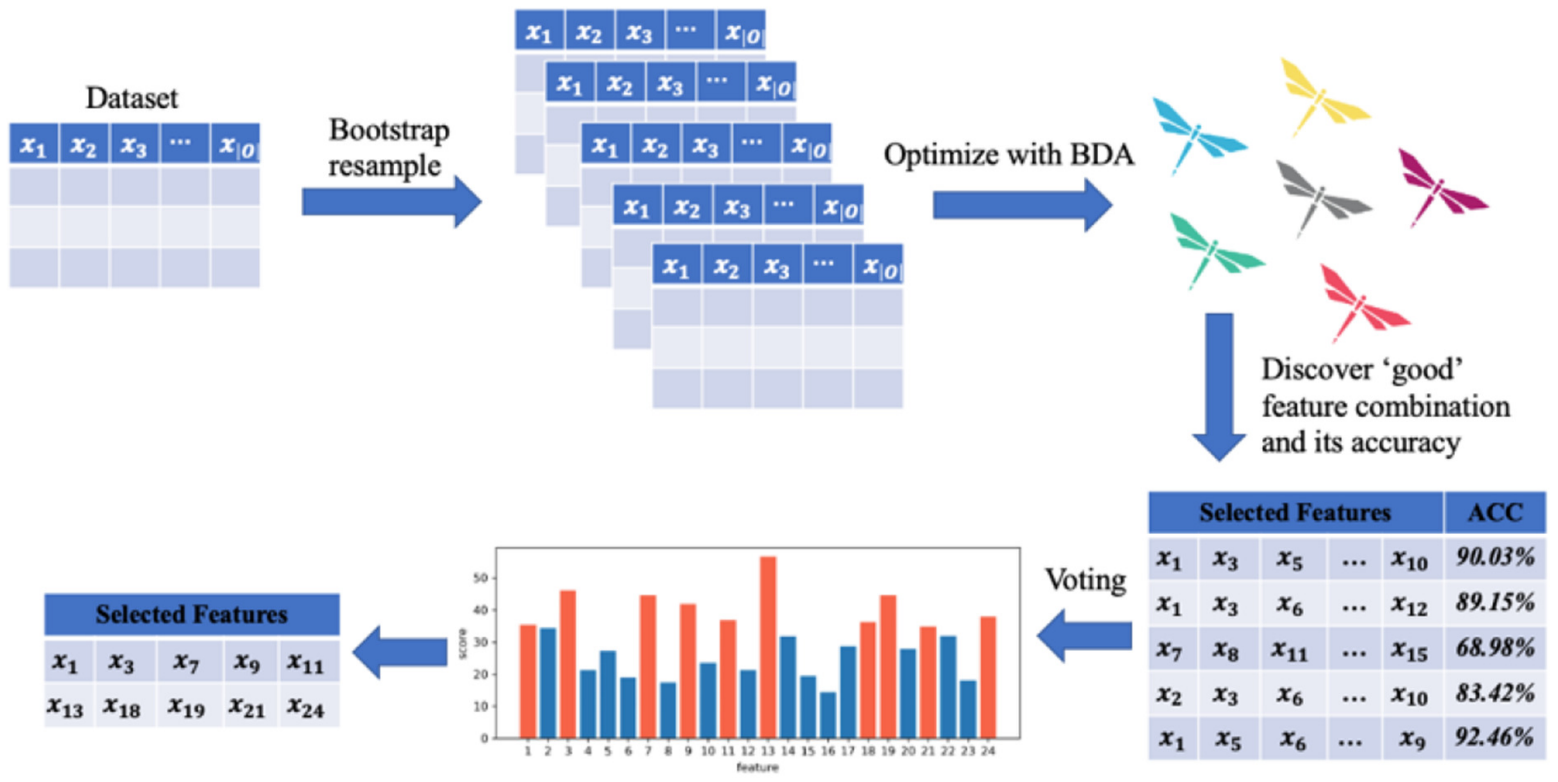
The flowchart of proposed SVM based feature selector.

In order to select key features from given data sets, a novel SVM-based selector is designed in this section, and its brief flowchart is given in Figure 3. The most important thing is to define “good” as the selected combination of features. A set of “good” features needs to have the following three characteristics. First, it can minimize the error of the model on the validation set. Second, the model obtained from this set of features has better robustness to the data set, that is, it is not easy to be attacked by individual outlier samples. Finally, choose as few metrics as possible. Combining the above requirements, we define the following objective function:


(10) $$\begin{array}{l} O\left(S,data\right)=\left[\frac{1}{K}\sum Err\left(S,data_{i}\right)\right]e^{-\frac{\left|S\right|}{\left|O\right|}}, \end{array}$$

where *data*_*i*_ is a sub-dataset obtained by resampling the original dataset data by *K*-fold cross-validation method. The SVM is used as the base classifier and training with the training data, *Err*(*S, data*_*i*_) is the prediction error on the corresponding validation set. *S* is the selected feature and |*S*| represents the number of *S*. |*O*| means the number of all candidate features. Then, the feature selection task is transformed into finding a combination of features that minimizes the objective function. This optimization problem can be implemented by BDA, that is, expand *S* with one-hot encoding as Ŝ, which is an |*O*|-dimensional vector, each element in Ŝ is 0 or 1, where Ŝ_*i*_ = 0 means that *S* does not select the feature *x*_*i*_, otherwise Ŝ_*i*_ = 1 means *S* contains a feature *x*_*i*_. Considering Ŝ as the position of the dragonfly, BDA updates Ŝ by imitating the movement trajectory of the dragonfly. We gave the pseudo-code of this feature selection algorithm in Algorithm 1.

**Algorithm 1:** SVM-based feature selection.

Input:	$dataset=\left\{X_{i}^{j},Y_{j}\right\}$, threshold *KK* for
	iterations and *EE* for object function;
Step 1.	Resample the dataset and obtain *m* sub-datasets $dataset_{m}=\left\{X_{i,m}^{j},Y_{j,m}\right\}$. Randomly generate a set of feature indicators $S^{l}=\left\{S_{i}^{l}\right\}$, and initialization *kk* = 0.
Step 2.	Calculate the object function $O_{m}\left(S^{l},\;dataset_{m}\right)$ by Equation (10).
Step 3.	Update the feature indicator *S*^*l*^ by Equation (9), and record the number of iterations *kk* = *kk*+1.
Step 4.	If: *kk* < *KK* and *O*_*m*_ > *EE*, Return to Step 2. Else: Jump out of the iteration and go to Step 5.
Step 5.	Obtain the local best feature indicator $\bar{S_{m}}=O_{m}(S^{l},\;dataset_{m})$.
Step 6.	Calculate the accuracy *Sc*_*m*_ of tenfold cross validation on dataset $\left\{\bar{S_{m}}X_{i}^{j},Y_{j}\right\}$, and calculate the average number of selected features $M=\frac{1}{m}|\bar{S_{m}}|$.
Step 7.	Local best feature indicator $\bar{S_{m}}$ selects the features by voting $SV=\underset{m}{\sum }\left(Sc_{m}•\bar{S_{m}}\right)$.
Step 8.	Find the *M* components with the highest votes in *SV*, and let $S_{i}=\{\begin{array}{c} 1,\;\;SV_{i}\in SD\\ 0,,\;\;SV_{i}\notin SD \end{array}.$
Output:	Selected Feature indicators *S* = {*S*_*i*_}.


In addition, the experiments are independently repeated on multiple sub-datasets through the bootstrapping sampling technique, making the selected features more robust. Denote the variable combination that minimizes the objective function on the sub-dataset *data*^*i*^ as *S*^*i*^, and the average classification accuracy on *K*-fold cross-validation is *Acc*^*i*^. A voting algorithm is used to add up the features selected on each sub-data set. In fact, these votes are not equal. The weight of each vote is determined by *Acc*^*i*^, which means that the combination of selected variables that makes the classification accuracy higher will be more favored. Finally, our SVM-based selector can obtain the *M* metrics with the most votes, and default *M* = *mean*(|*S*^*i*^|), which is the average number of selected features in sub-datasets, to avoid adding more hyperparameters.

## Result

### Sample characteristics

Ultimately, we included 186 adolescents with mood disorders, 137 of whom had NSSI behaviors and 49 of whom did not. We also enrolled 96 typically developing adolescents. A total of 77 males and 205 females were included in this study, with a male-to-female ratio of ~1:3, which was close to the male-to-female incidence ratio of NSSI (65). Comparisons between groups revealed that the differences between the three groups were statistically significant in terms of adolescent age, education level, and parental age, while differences in parental education levels were not significant. Sociodemographic characteristics were shown in Table 1.

**Table 1:** Sociodemographic characteristics of participants.

	**Mood disorder group**		**TD group (*n* = 96)**	***x*^2^/*F* (*P*-value)**
	**With NSSI group (*n* = 137)**	**Without NSSI group (*n* = 49)**		
Gender (*n*, %)				9.936 (0.019)
Male	27 (19.70)	18 (36.73)	32 (33.33)	
Female	110 (80.29)	31 (63.27)	64 (66.67)	
Age (years, mean ± SD)	15.569 ± 2.244	17.265 ± 3.205	20.375 ± 3.854	83.872 (<0.001)
Ethnic (*n*, %)				10.316 (0.006)
Han nationality	117 (85.40)	46 (93.88)	93 (96.88)	
Others	20 (14.60)	3 (6.12)	3 (3.12)	
Adolescent education level (*n*, %)				39.728 (<0.001)
≤ Middle school	5 (3.65)	1 (2.04)	4 (4.17)	
High school	55 (40.16)	12 (24.49)	6 (6.25)	
≥College	77 (56.20)	36 (73.47)	87 (89.58)	
Mother age (years, mean ± SD)	42.693 ± 4.736	44.408 ± 4.651	46.833 ± 5.625	34.497 (<0.001)
Father age (years, mean ± SD)	45.503 ± 4.710	48.346 ± 4.401	49.198 ± 5.417	29.549 (<0.001)
Mother education level (*n*, %)				3.206 (0.524)
≤ Middle school	64 (46.72)	18 (36.73)	50 (52.08)	
High school	42 (30.66)	17 (34.69)	25 (26.04)	
≥College	31 (22.62)	14 (28.58)	21 (21.88)	
Father education level (*n*, %)				1.111 (0.893)
≤ Middle school	56 (40.88)	18 (36.73)	40 (41.67)	
High school	43 (31.39)	16 (32.65)	33 (34.38)	
≥College	38 (27.73)	15 (30.62)	23 (23.95)	

NSSI, non-suicidal self-injury.

### Groups differences in influencing factors

Our result showed that with NSSI group scored highest on all psychological/behavioral problems except for the sexual abuse score, followed by without NSSI and TD groups. The non-parametric Kruskal-Wallis tests revealed significant differences between the three groups for psychological and behavioral variables, including anxiety, depression, personality traits, emotion regulation ability, and childhood trauma (Table 2). Specifically, the average anxiety and depression levels were moderate to severe (>14) and severe (>19) in the mood disorder with NSSI group, moderate (>10) and moderate to severe (>15) in the mood disorder without NSSI group, and mild in the TD group. Furthermore, the PDQ-4+ scores indicated that adolescents in the mood disorder with NSSI group may have paranoid (4.175 ± 1.499 >4), borderline (7.700 ± 1.858), avoidant (6.401 ± 1.572), and obsessive-compulsive (6.182 ± 2.381) personality traits, whereas adolescents in the mood disorder without NSSI group may have borderline (6.041 ± 2.267), avoidant (5.918 ± 1.923), and obsessive-compulsive (5.592 ± 2.571) personality traits, while the TD group did not indicate any personality traits. *Post-hoc* pairwise tests showed that compared with the mood disorder with NSSI group, the mood disorder without NSSI group scored significantly lower on PHQ-9, borderline, physical abuse, awareness, clarity, and impulse scores; the TD group scored significantly lower on all study variables except for sexual abuse score. Moreover, compared with the mood disorder without NSSI group, the TD group scored significantly lower on all study variables except for physical abuse and neglect, sexual abuse, and awareness. Furthermore, when the mood disorder (both with and without NSSI) and TD groups were compared, there were significant differences between the two groups on all indicators except sexual abuse (Table 2).

**Table 2:** Groups differences and pairwise comparison of influencing factors.

	**Mood disorder group**		**TD group (*n* = 96)**	***x*^2^/*F* (*P*-value)**	***Post-hoc* comparison**
	**With NSSI group (*n* = 137)**	**Without NSSI group (*n* = 49)**			
GAD-7	14.088 ± 4.733	12.571 ± 5.410	4.865 ± 4.127	124.344 (<0.001)	With NSSI > without NSSI ^a^, with NSSI > TD, without NSSI > TD, mood disorder > TD
PHQ-9	19.015 ± 5.277	15.939 ± 5.898	5.875 ± 4.837	153.116 (< 0.001)	With NSSI > without NSSI > TD, mood disorder > TD
**PDQ-4+**					
Paranoid	4.175 ± 1.499	3.347 ± 1.985	2.312 ± 1.932	48.757 (<0.001)	With NSSI > without NSSI ^a^, with NSSI > TD, without NSSI > TD, mood disorder > TD
Borderline	7.700 ± 1.858	6.041 ± 2.267	2.635 ± 2.377	143.956 (<0.001)	With NSSI > without NSSI > TD, mood disorder > TD
Histrionic	4.292 ± 1.919	4.041 ± 2.04	3.010 ± 2.472	19.821 (<0.001)	With NSSI > without NSSI ^a^, with NSSI > TD, without NSSI > TD, Mood disorder > TD
Avoidant	6.401 ± 1.572	5.918 ± 1.923	2.864 ± 2.737	87.707 (<0.001)	With NSSI > without NSSI ^a^, with NSSI > TD, without NSSI > TD, mood disorder > TD
Obsessive-compulsive	6.182 ± 2.381	5.592 ± 2.571	3.667 ± 2.592	46.231 (<0.001)	With NSSI > without NSSI ^a^, with NSSI > TD, without NSSI > TD, mood disorder > TD
**CTQ-SF**					
Physical abuse	7.043 ± 3.035	6.367 ± 3.474	5.427 ± 1.068	33.958 (<0.001)	With NSSI > without NSSI, with NSSI > TD, without NSSI > TD ^a^, mood disorder > TD
Emotional abuse	12.372 ± 4.408	11.041 ± 4.811	6.719 ± 2.192	111.007 (<0.001)	With NSSI > without NSSI ^a^, with NSSI > TD, without NSSI > TD, mood disorder > TD
Sexual abuse	5.700 ± 0.428	5.449 ± 1.566	5.635 ± 1.621	2.076 (0.354)	With NSSI > without NSSI > TD ^a^, mood disorder > TD ^a^
Physical neglect	9.518 ± 3.163	8.775 ± 3.039	8.364 ± 2.884	8.302 (0.016)	With NSSI > without NSSI ^a^, with NSSI > TD, without NSSI > TD ^a^, mood disorder > TD
Emotional neglect	17.212 ± 5.448	15.327 ± 4.963	11.958 ± 5.714	45.306 (<0.001)	With NSSI > without NSSI ^a^, with NSSI > TD, without NSSI > TD, mood disorder > TD
**DERS**					
Awareness	18.124 ± 4.901	15.898 ± 4.301	15.958 ± 4.085	14.445 (0.001)	With NSSI > without NSSI, with NSSI > TD, without NSSI > TD ^a^, mood disorder > TD
Clarity	15.146 ± 3.561	13.469 ± 4.300	11.385 ± 3.094	55.570 (<0.001)	With NSSI > without NSSI > TD, mood disorder > TD
Non-acceptance	19.650 ± 5.578	18.122 ± 5.861	12.917 ± 4.936	70.120 (<0.001)	With NSSI > without NSSI ^a^, with NSSI > TD, without NSSI > TD, mood disorder > TD
Impulse	21.036 ± 6.227	17.918 ± 6.452	12.708 ± 5.216	81.967 (<0.001)	With NSSI > without NSSI > TD, mood disorder > TD
Goals	20.139 ± 3.960	19.163 ± 4.524	14.063 ± 4.460	81.577 (<0.001)	With NSSI > without NSSI ^a^, with NSSI > TD, without NSSI > TD, mood disorder > TD
Strategies	30.766 ± 6.217	27.898 ± 7.092	18.739 ± 7.325	108.033 (<0.001)	With NSSI > without NSSI ^a^, with NSSI > TD, without NSSI > TD, mood disorder > TD
Total	124.861 ± 20.890	112.469 ± 21.396	85.770 ± 21.879	114.391 (<0.001)	With NSSI > without NSSI > TD, mood disorder > TD

NSSI, non-suicidal self-injury; TD, typical developmental; GAD-7, Generalized Anxiety Disorder-7; PHQ-9, Patient Health Questionnaire-9; PDQ-4+, Personality Diagnostic Questionnaire-4+; CTQ-SF, Childhood Trauma Questionnaire-Short Form; DERS, Difficulties in Emotion Regulation Scale.

Mean ± SD.

^a^*P* > 0.05.

### Identify adolescents with the mood disorder

**Figure 4:**
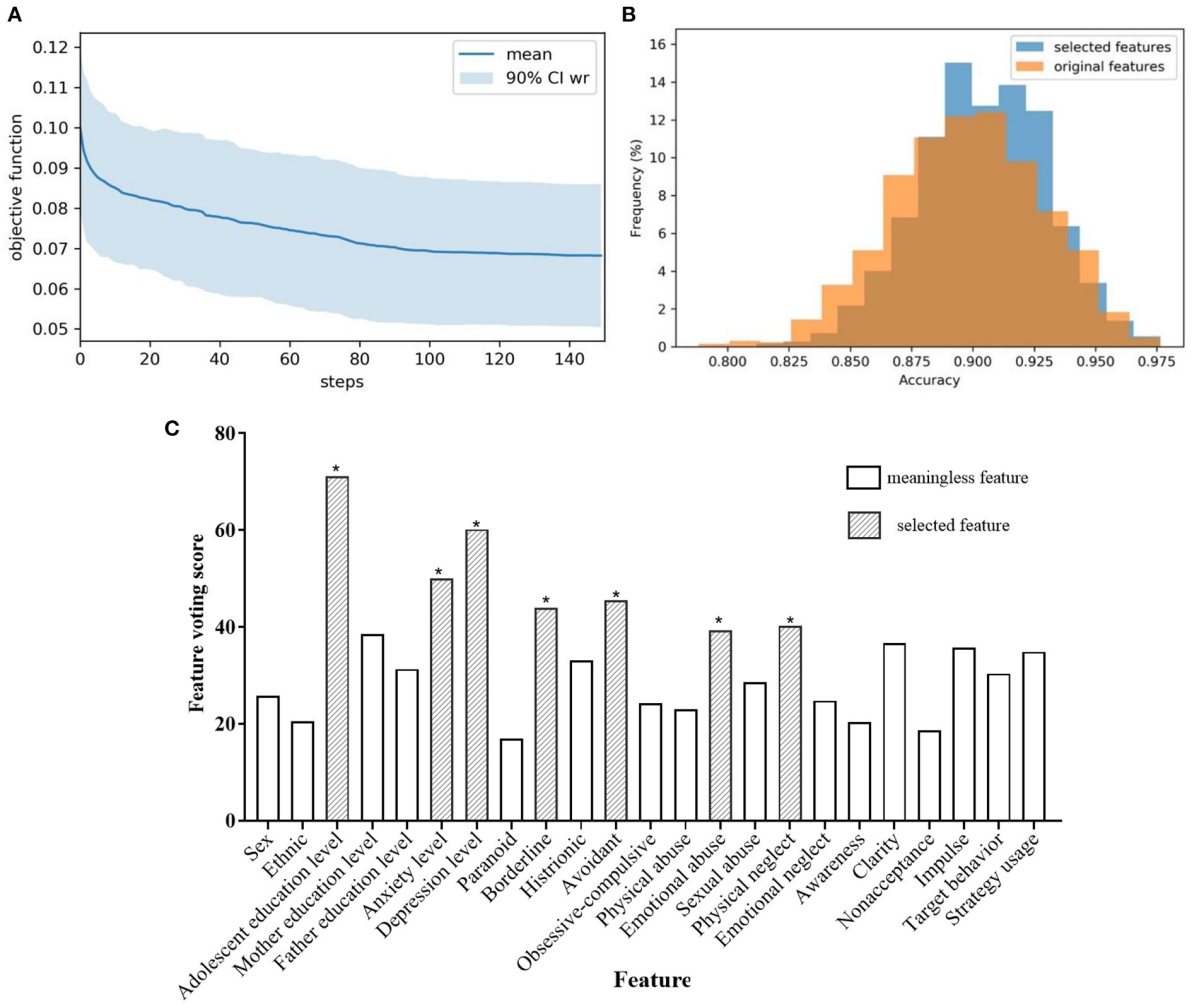
**(A)** Objective function of the training process for identifying mood disorders; **(B)** The distribution of classification accuracy for identifying mood disorders; **(C)** Feature voting for identifying mood disorders; *selected feature.

Since adolescents with NSSI are often accompanied by mood disorders, identifying indicators that distinguish mood disorders from TD adolescents lays the foundation for further prediction of NSSI behavior. Hyperparameters in the SVM-based selector were set to default, with penalty co-efficient *C* = 1. The 10 dragonfly positions were randomly initialized in BDA, the personal learning rate was set as 0.4, and the group learning rate was 0.7. The objective function gradually decreased with the training process and converged after 150 iterations, and the shaded area represented the variance of 100 independent replicates (Figure 4A). The average number of selected features was 7.17, and the votes for each feature were plotted in Figure 4C.

It could be seen that adolescent education level, anxiety, and depression level, borderline and avoidant personality disorder, emotional abuse, and physical neglect were the seven characteristics that received the most votes on the 100 sub-datasets, and were thought to be highly correlated with identifying mood disorders (Figure 4C). To further illustrate the effectiveness of the selector, we verified the classification performance on the resampling sub-dataset using all features and filtered features, respectively. The distribution of classification accuracy was depicted in Figure 4B, which showed that only seven features were selected, but the prediction accuracy was even slightly improved, indicating that using only these selected features was sufficient to distinguish the mood disorder group from the TD group while avoiding the interference of irrelevant variables. We also compared different classification algorithms (Table 3), which demonstrated that SVM performed better in classification tasks. Most machine learning models could accurately distinguish between the mood disorder and TD groups, whereas LASSO got disoriented by failing to describe non-linear associations. Random forests implied feature selection and achieved better results than KNN and SVM when all original features were used.

**Table 3:** Average classification accuracy of different algorithms for identifying mood disorders.

**Algorithms**	**Accuracy**
K-nearest neighbor	89.44%
Logistic regression	86.50%
LASSO	88.41%
Elastic net	77.65%
Ridge regression	75.75%
Decision tree	84.96%
Random forests	89.79%
SVM with all features	89.28%
SVM with selected features	**90.60%**

LASSO, least absolute shrinkage and selection operator; SVM, support vector machine.

### Identify adolescents with NSSI

**Figure 5:**
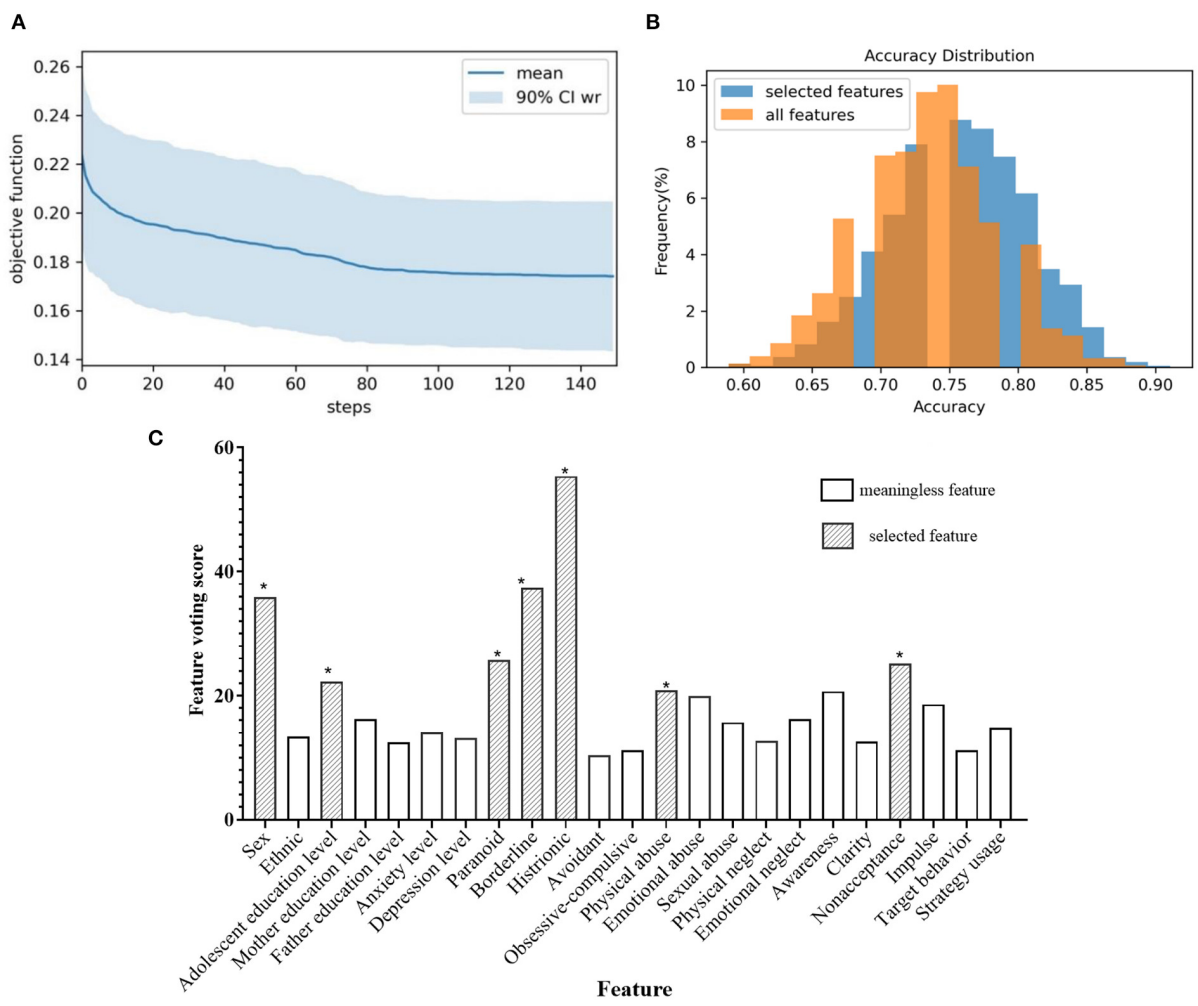
**(A)** Objective function of the training process for identifying NSSI; **(B)** The distribution of classification accuracy for identifying NSSI; **(C)** Feature voting for identifying NSSI; *selected feature.

The second part was to discover key factors in identifying NSSI. Similar to the mood disorders above, an SVM-based feature selector was optimized with BDA for 150 steps, and the decay of the objective function was plotted in Figure 5A. We also calculated the voting in 100 independent replicates (Figure 5C), where gender, adolescent education level, paranoid, borderline, and histrionic personality disorders, physical abuse, and non-acceptance of emotional responses were highlighted in red. Females were more likely to have NSSI than males, and the occurrence of NSSI behaviors was negatively correlated with their educational level. Special personality traits (e.g., paranoid, borderline, and histrionic) can help distinguish between NSSI behavior in patients with mood disorders. We demonstrated that using the selected seven key indicators to predict the occurrence of NSSI behavior was more accurate than using all indicators, proving the rationality of feature selection (Figure 5B).

We also compared other machine learning approaches to distinguishing NSSI from mood disorders (Table 4). Distinguishing mood disorders in adolescents with and without NSSI was a more difficult task, and the accuracy of all machine learning algorithms we considered was about 66–76%, although feature selection still improved prediction precision. In 1,000 independent repeated experiments, the average accuracy of SVM on the original features was 73.45%, with the highest accuracy of 87.39%, which was higher than KNN and Logistic regression, indicating that SVM retained some advantages in high-dimensional non-linear classification tasks. Our experiments also showed that the decision tree model was prone to overfitting, resulting in poor classification accuracy. The random forest model avoided this problem by constructing multiple decision trees. In addition, the random forest used important features instead of all features for prediction by limiting the number of nodes, and the regularization term in LASSO also played a role in feature selection, which had a better performance than the SVM method using all features. Our SVM-based method combined the above two advantages, which not only retained the high classification accuracy of SVM, but also avoided the attack of unimportant or harmful variables on the prediction model. Compared with other machine learning methods, the average accuracy was improved by 1%, and the highest accuracy could exceed 90%.

**Table 4:** Average classification accuracy of different algorithms for identifying NSSI.

**Algorithms**	**Accuracy**
K-nearest neighbor	72.51%
Logistic regression	73.01%
LASSO	74.25%
Elastic net	64.50%
Ridge regression	66.64%
Decision tree	73.08%
Random forests	74.91%
SVM with all features	73.45%
SVM with selected features	**75.74%**

LASSO, Least absolute shrinkage and selection operator; SVM, support vector machine.

## Discussion

In this paper, we proposed an SVM-based feature selection method for identifying influencing factors on adolescents with NSSI. An objective function was designed to describe how “good” the feature combinations, which required both higher accuracy and fewer features. Bootstrapping sampling made the features obtained by the selector more robust and might not be disturbed by abnormal samples. In addition, our findings proved that using only a few selected features can achieve higher accuracy than using all features, indicating that these selected features were critical for accurate classification, and that the unselected features interfered with the model. Furthermore, as a powerful machine learning technique, SVM exhibited better performance than other statistical methods. Our study showed that adolescent's anxiety and depression levels, borderline and avoidant personality traits, experiencing emotional abuse and physical neglect in childhood, and education level were associated with mood disorders in adolescents; adolescent's gender, paranoid and histrionic personality traits, suffering physical abuse in childhood, emotion non-acceptance, and their education level were associated with an increased risk of NSSI.

We found that adolescents in the mood disorder with NSSI group scored higher on psychological symptoms and childhood trauma than the without NSSI and TD groups, while the mood disorder group scored significantly higher on almost variables than the TD group. These findings supported the point that there were significant differences in psychological behavior between adolescents with and without NSSI. Not only that, adolescents with and without NSSI with mood disorders shared similar psychological and behavioral characteristics, but these characteristics were more prominent in adolescents with NSSI. Overall, adolescents with NSSI and mood disorders have similar but independent risk factors. However, in the overall sample of this study, univariate analyzes found no statistically significant differences in most variables between the mood disorder groups with and without NSSI.

In this study, the reporting rate of NSSI in female adolescents was higher than that in male adolescents, and multivariate analysis also showed that male was a protective factor for NSSI behavior in adolescents, which was consistent with the results of a recent meta-analysis (3). Previous studies have shown that the gender difference in clinical samples was larger than that in community samples, and the difference gradually decreases with age. In addition, there were differences in the patterns and motivations of NSSI among adolescents of different genders (66). Females were more likely to engage in cutting, scratching, and biting as means of NSSI, while males were more likely to burn, hit and bang. Moreover, female adolescents engaged in NSSI mainly for emotional regulation and self-control, while male adolescents were more eager to generate impulsive pleasure. The reasons for gender differences in adolescent NSSI may include (3): (a) biological factors: hormonal (e.g., androgens and estradiol) differences between males and females may influence gender involvement in NSSI; (b) differences in male and female emotion regulation strategies: research has shown that females were more likely than males to engage in emotion regulation strategies, and NSSI was considered an emotion regulation strategy.

The results of both univariate and multivariate analyzes showed that anxiety and depression levels were associated with NSSI in adolescents, which was consistent with previous research. Foreign studies have reported that there was a bidirectional correlation between anxiety, depression and NSSI. The higher the score of the anxiety and depression scale, the greater the likelihood of NSSI. Conversely, NSSI will increase anxiety and depression (67). Using a latent growth curve modeling, scholars suggested that lifetime depression predicted the longitudinal course of NSSI from grade 10 to 12, with depressed adolescents showing greater and more stable NSSI engagement over time than non-depressed adolescents (68). In addition, studies have shown the mediating role of depression on other factors (i.e., peer acceptance and frequent nightmares) and NSSI behavior in adolescents (69, 70). Furthermore, post-traumatic stress disorder, dissociative disorders, obsessive-compulsive disorders, eating disorders, sleep disorders, and substance use disorders were also common co-occurring disorders in adolescents with NSSI (1, 71).

Adolescent NSSI is associated with specific personality traits. Personality is a stable and lasting characteristic of a person's mental activity, especially in emotional activity and volitional behavior. Individuals with personality disorders were frequently diagnosed as being at risk for suicide, which suggested that personality pathology may reflect important individual differences in predicting suicide attempts (72). Previous studies showed that several personality disorder dimensions (i.e., paranoid, antisocial, borderline, histrionic, and dependent) emerged as risk factors for suicidal attempts based on univariate models (72). However, Jenkins et al. (72) found borderline personality disorder severity uniquely predicted suicidal attempts over other personality disorder severity based on multivariate models, which was similar to our findings. Specifically, borderline personality disorder is one of the most common co-morbidities of adolescent NSSI (71). Studies have shown that ~61% of adolescents with borderline personality disorder engaged in at least one NSSI behavior (73). More than that, NSSI was considered a precursor to the development of borderline personality disorder under the sociobiological developmental model (16). A review based on seven longitudinal studies showed a longitudinal association between NSSI and borderline personality disorder symptoms in adolescents (16).

Furthermore, emotion regulation was one of the motivations for NSSI (74), which was consistent with our findings. The previous study has demonstrated that emotion regulation ability is negatively associated with NSSI behavior (75). In adolescence, adaptive internal emotion regulation has limited efficacy; therefore, adolescents lack effective coping strategies in dealing with negative emotions and are more likely to adopt NSSI behaviors to alleviate negative emotions, which results in impaired social relationships and increased negative emotions, forming a vicious cycle. Emotional regulation ability is developed during the emotional interactions with caregivers in the early stage of children's growth. Parents' denigration or contempt behaviors and frequent negative emotions against children will weaken children's emotional regulation ability and disrupt the development of normal emotional regulation ability (76). Therefore, effective emotion regulation strategies should be carried out based on adolescents and their parents in order to achieve a virtuous cycle of emotion regulation.

Another important finding was that physical abuse in childhood significantly increased the risk of NSSI in adolescents. Studies have demonstrated that childhood abuse and neglect (both physical and emotional) were positively associated with self-injurious behavior and that the incidence of NSSI increased with greater levels of abuse and neglect (77, 78). The American scholar demonstrated that childhood physical and sexual abuse was strongly associated with adolescent NSSI and that the frequency of NSSI increased with the frequency of abuse (79). Moreover, the incidence of NSSI was higher in females than in males when exposed to high levels of sexual abuse, emotional neglect, and physical abuse (80). However, sexual abuse did not show significance in our study. Furthermore, we speculated that since China has fully implemented 9-year compulsory education, educational attainment to some extent implies the age of adolescents. Due to NSSI, they may interrupt their studies, resulting in lower educational attainment. Therefore, parents and health managers should pay attention to the healthy growth of children and reduce the occurrence of childhood abuse and trauma.

Our research focused on improving people's mental health based on artificial intelligence technology and discovered key indicators that affect NSSI in adolescents. Unlike univariate model analysis, we used a multivariate model to explore the risk factors, which better revealed the interactions between factors. However, sampling methods may limit the generalizability of study results. The majority of participants were outpatients, and the results may differ from inpatients or non-treatment seeking populations. Moreover, this study was cross-sectional, which could not infer the causal relationships between factors and NSSI behaviors in adolescents with mood disorders. Data from this survey were obtained from questionnaires and recall bias was unavoidable. Future studies could use a longitudinal study design to follow up on risk factors for adolescent NSSI behavior. It is worth noting that from family promotion to collective or company, our proposed SVM-based selector can also be used as a data-driven technique to improve the mental health of members and employees, find the key causes of employee mental health problems, and help companies reduce possible employee mental health problems.

## Data availability statement

The raw data supporting the conclusions of this article will be made available by the authors, without undue reservation.

## Ethics statement

The studies involving human participants were reviewed and approved by the Medical Ethics Committee of the Second Xiangya Hospital of Central South University (MD20200309). Written informed consent to participate in this study was provided by the participants' legal guardian/next of kin.

## Author contributions

JY and YL conceived and designed the research. YC, GY, and ZW analyzed the data. XF and YT collected the data. JY and YC reviewed and edited the manuscript. All authors contributed to the article and approved the submitted version.

## Funding

This study was supported by the Clinical Nursing Research Foundation of the Second Xiangya Hospital of Central South University (grant number 2021-HLKY-05), Major Scientific and Technological Projects in Hunan Province (grant number 2020SK208), the National Natural Science Foundation of China (grant numbers 81974217 and 81901388), the Natural Science Foundation of Hunan Province, China (grant numbers 2020JJ5825 and 2020JJ5830), and the Major Scientific and Technological Projects for Collaborative Prevention and Control of Birth Defects in Hunan Province (grant number 2019SK1015).

## Conflict of interest

The authors declare that the research was conducted in the absence of any commercial or financial relationships that could be construed as a potential conflict of interest.

## Publisher's note

All claims expressed in this article are solely those of the authors and do not necessarily represent those of their affiliated organizations, or those of the publisher, the editors and the reviewers. Any product that may be evaluated in this article, or claim that may be made by its manufacturer, is not guaranteed or endorsed by the publisher.
